# PReventing early unplanned hOspital readmission aFter critical ILlnEss (PROFILE): protocol and analysis framework for a mixed methods study

**DOI:** 10.1136/bmjopen-2016-012590

**Published:** 2016-06-28

**Authors:** Timothy S Walsh, Lisa Salisbury, Eddie Donaghy, Pamela Ramsay, Robert Lee, Janice Rattray, Nazir Lone

**Affiliations:** 1 University Department of Anaesthesia, Critical Care, and Pain Medicine, School of Clinical Sciences, University of Edinburgh, Edinburgh, UK; 2 MRC Centre for Inflammation Research, University of Edinburgh, Edinburgh, UK; 3 Usher Institute, Centre for Population Health Sciences, University of Edinburgh, Edinburgh, UK; 4 Department of Nursing Studies, University of Dundee, Dundee, UK

**Keywords:** QUALITATIVE RESEARCH, REHABILITATION MEDICINE

## Abstract

**Introduction:**

Survivors of critical illness experience multidimensional disabilities that reduce quality of life, and 25–30% require unplanned hospital readmission within 3 months following index hospitalisation. We aim to understand factors associated with unplanned readmission; develop a risk model to identify intensive care unit (ICU) survivors at highest readmission risk; understand the modifiable and non-modifiable readmission drivers; and develop a risk assessment tool for identifying patients and areas for early intervention.

**Methods and analysis:**

We will use mixed methods with concurrent data collection. Quantitative data will comprise linked healthcare records for adult Scottish residents requiring ICU admission (1 January 2000–31 December 2013) who survived to hospital discharge. The outcome will be unplanned emergency readmission within 90 days of index hospital discharge. Exposures will include pre-ICU demographic data, comorbidities and health status, and critical illness variables representing illness severity. Regression analyses will be used to identify factors associated with increased readmission risk, and to develop and validate a risk prediction model. Qualitative data will comprise recorded/transcribed interviews with up to 60 patients and carers recently experiencing unplanned readmissions in three health board regions. A deductive and inductive thematic analysis will be used to identify factors contributing to readmissions and how they may interact. Through iterative triangulation of quantitative and qualitative data, we will develop a construct/taxonomy that captures reasons and drivers for unplanned readmission. We will validate and further refine this in focus groups with patients/carers who experienced readmissions in six Scottish health board regions, and in consultation with an independent expert group. A tool will be developed to screen for ICU survivors at risk of readmission and inform anticipatory interventions.

**Ethics and dissemination:**

Data linkage has approval but does not require ethical approval. The qualitative study has ethical approval. Dissemination with key healthcare stakeholders and policymakers is planned.

**Trial registration number:**

UKCRN18023.

**Strengths and limitations of this study**

- A concurrent mixed methods study using both quantitative and qualitative data to understand factors influencing unplanned readmissions, with a strong focus of patient and carer perspectives.
- Use of national (Scotland-wide) data for the deductive quantitative analysis, and interviews/focus groups across multiple geographical regions for the inductive qualitative analyses, thereby increasing the generalisability of findings at least within the UK.
- A pre-defined strategy for data integration that includes independent expert review at key stages, and further consultation with patients and carers for confirmation of key findings.
- Statistical modelling of quantitative data is limited by the variables available within national linked data sets.
- Findings may not be generalisable to other healthcare systems or intensive care populations.

## Introduction

The disabilities and impairments that follow critical illness include physical, psychological and cognitive decline, and have been termed the post-intensive care unit (ICU) syndrome (PICS).^1^ Impact is also high for families/carers, especially in social and psychological domains.^2–5^ We recently found >50% of ICU survivors have two or more pre-existing comorbidities,^6^ ^7^ and many acquire new functional disability associated with critical illness.^8^ New psychological morbidity is especially prevalent (typically >20%).^9–11^ A ‘step change’ deterioration of cognition and activities of daily living (ADLs) is common, especially for older patients with pre-existing impairments.^8^ ^12^ Unsurprisingly, health-related quality of life (HRQoL) is significantly reduced and often fails to match population norms.^13^ Pre-existing comorbidity and frailty are associated with lower HRQoL, function and mortality among ICU survivors.^6^ ^14–17^ Compared with matched controls, critical illness generates 10% excess 5-year mortality; although absolute mortality rates are highest for older patients with comorbidity, the greatest excess mortality compared with control populations occurs in younger patients.^6^

Published reviews summarise the range and severity of PICS disability.^1^ Prevalent physical symptoms include fatigue and weakness, which contribute to markedly reduced functional status and ability to perform ADLs. Reviews of intervention research (including a recently completed Cochrane review) found few studies of generally low quality, and lack of clear evidence for interventions based on physical rehabilitation strategies in isolation.^18^ ^19^ Strongest evidence was for a strategy supporting self-management.^20^ The optimum approach for reducing psychological morbidity is unknown, with only weak evidence for organised follow-up or the use of diaries.^21^ ^22^ ICU survivors report multidimensional needs, extending beyond biomedical models of recovery.^23^ Patients seek information and support to adjust to new disability and re-engage with independent living.^24–26^ Future interventions need to consider the interplay of physical, psychological, cognitive and social factors in order to address the multiple factors contributing to poor recovery.

There are few studies of resource use following critical illness, but a systematic review indicated high direct healthcare costs post-ICU discharge.^27^ In a recent UK-based randomised trial, mean 12-month secondary care costs post-ICU discharge were £49k.^7^ A national data linkage cohort study in Scotland restricted to postindex hospital admission found acute hospital costs were 50% greater over 5 years than matched non-ICU hospital cohorts (£14k per patient).^6^ The major cost driver was unplanned acute hospital readmissions, which occurred in 40% of older survivors by 6 months.

Unplanned hospital readmission is widely used as a quality indicator but has not been studied following critical illness. The health literature suggests that multiple factors contribute, including multimorbidity, functional impairments, the nature and severity of illness, high-risk medications and polypharmacy.^28^ ^29^ Organisational issues are also important, including premature/inadequate discharge planning, inadequate communication across acute and primary care settings, absence of rehabilitative support, poor engagement of family members in discharge planning and postdischarge care, and absent/delayed follow-up. Social factors include age, lower socioeconomic/educational status and poor health literacy. Some risks may be modifiable through anticipatory care models. For example, identifying patients at highest risk prior to hospital discharge could enable targeted resource and support during the early postdischarge period. Understanding the drivers for readmission could inform the content, structure and timing of preventive strategies. Given the high costs associated with unplanned readmissions following critical illness, evidence-based interventions could be cost-effective by reducing hospital costs and improving HRQoL in the community.

## Study aims

The aims of the *PR*eventing early unplanned h*O*spital readmission a*F*ter critical *IL*ln*E*ss (PROFILE) study are:

- to understand the risk factors associated with early (90-day) readmission among ICU survivors;
- to develop a risk model to identify ICU survivors at highest risk of early readmission before they are discharged from hospital;
- to understand the reasons ICU survivors require early readmission, including modifiable and non-modifiable factors;
- to develop an evidence-based taxonomy/construct to screen patients at risk of readmission and identify areas for early intervention.

The final outputs of the project are intended to be a toolkit for hospitals/health boards that could drive quality improvement for the ICU survivors' recovery journey including a risk assessment tool, and a checklist to direct interventions that may minimise readmission risk.

Our specific research questions (RQs) are:

- RQ1: What are the risk factors prior to hospital discharge associated with early unplanned readmission to acute hospital in ICU survivors?
- RQ2: Can ICU survivors at highest risk of subsequent readmission be identified before hospital discharge?
- RQ3: What are the reasons early unplanned readmissions occur?
- RQ4: What factors are potentially modifiable through early intervention?

## Conceptual framework

**Figure 1:**
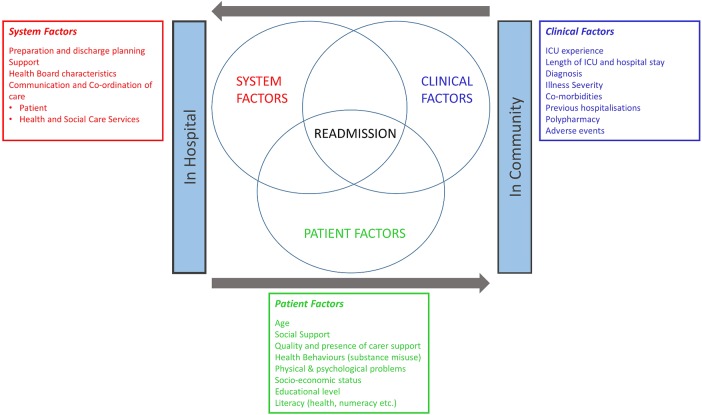
Conceptual framework for the range of factors that may contribute to unplanned hospital readmission following hospitalisation with an episode of critical illness. ICU, intensive care unit.

We have undertaken an integrative literature review of quantitative, qualitative and grey literature to identify potential risk factors for an unplanned acute hospital readmission for survivors of critical illness and for wider patient populations (PROSPERO registration: CRD42016019524). The review enabled the development of an initial conceptual framework of possible drivers for unplanned readmission in the critical care survivor population (figure 1).

Three general risk categories were identified. These were systemic, patient-centred and clinical factors. Systemic factors included premature/poor-quality discharge planning, poor communication between healthcare staff across acute and primary care settings, insufficient engagement with patient and family members in discharge planning and postdischarge care, and insufficient/delayed follow-up care post hospital discharge.^28–32^ Patient-centred factors were poor social support, living alone or unmarried,^33^ ^34^ low socioeconomic/educational status, unemployed and poor health literacy.^30^ ^33^ ^35–37^ Key clinical factors were polypharmacy, use of high-risk medications, chronic illness, multimorbidity, functional impairment, mental illness and substance misuse.^28^ ^29^ ^38^ ^39^

## Study overview and methodological approach

PROFILE is using a mixed methods approach. We will link large healthcare databases available in Scotland which capture data that include all admissions to Scottish ICUs, all hospital admission episodes, diagnoses, deaths, and some prescribing and other data. Data will be used in quantitative modelling to address RQs1–3. Concurrent to these analyses, we will conduct interviews with patients and their carers who have required recent unplanned hospital readmission following a hospital episode that required ICU care. Qualitative analysis of interview transcripts will explore the common factors contributing to unplanned readmission from a patient/relative perspective.

The qualitative and quantitative studies will occur concurrently and will be complementary. Equal importance will be placed on data acquired from both sources. We will use regular triangulation of findings as they emerge at meetings of the study investigators to maximise the value of both data sets. For example, known or emerging predictors in quantitative analyses may influence themes explored in interviews; conversely, issues emerging from interviews may influence the choice of exposures/variables explored in quantitative analyses. In addition, we will invite an external independent expert group to comment on the development of the semistructured interview schedules for patients and carers/family members, emerging findings, the analytic approaches to data integration proposed, and provide additional advice to the study investigators from multiple perspectives. The expert group will meet at the start of the project (for orientation and to establish roles), once data collection is completed and preliminary analysis undertaken, and at the end of data integration.

**Figure 2:**
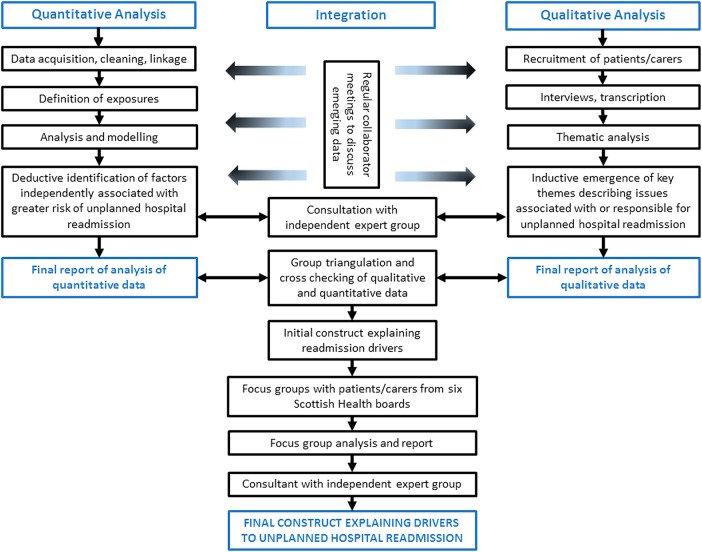
Flow diagram illustrating the overall structure of the PROFILE study. PROFILE, PReventing early unplanned hOspital readmission aFter critical ILlnEss.

A detailed separate qualitative and quantitative study report will be prepared. In addition, we will sequentially triangulate the mixed data sources to develop a final construct designed to capture the main drivers of unplanned readmission and how they may interplay. A series of focus groups with patients who required unplanned early readmission and their carers will be undertaken to explore the validity of the construct. These will take place in six Scottish health boards to ensure maximum national representation, and to decrease the chance of missing important region-specific information. Following any modification, the construct will be presented to the expert group for independent review and final validation. The final construct will aim to identify the key drivers of unplanned readmission and how they interact, and a taxonomy that could be operationalised as a checklist or screening tool within clinical practice. The overall structure of the research programme is illustrated in figure 2.

## Protocols and methodology for programme components

### Quantitative study

#### Study population, setting and databases

We will use a cohort study design. Data sources will be routinely collected, administrative, linked registries: Scottish Intensive Care Society Audit Group (SICSAG), Scottish Morbidity Record of acute hospital admissions (SMR01), Scottish death records, acute psychiatric hospital admissions (SMR04), Scottish Cancer Registry, Scottish Outpatient Registry (SMR00) and prescribing database. The SICSAG registry captures all adult general intensive care activity serving a population of around 5 million (4.2 million aged ≥16) within Scotland. The cohort will consist of Scottish residents aged ≥16 admitted to and discharged from general ICUs in Scotland between 1 January 2000 and 31 December 2013 who survived to hospital discharge (index admission). We will analyse these population-level data, which comprise a complete national cohort of the population at risk (survivors of an episode of critical illness discharged from the acute hospital). This study gained approval from the Privacy Advisory Committee of NHS National Services Scotland. The Research Ethics Committee granted a waiver. All data will be anonymised prior to release to the researchers.

#### Analytic approaches

*RQ1: What are the risk factors prior to hospital discharge associated with early unplanned readmission to acute hospital in ICU survivors?* We will identify the patient demographic, prehospital and acute illness factors most strongly associated with early readmission. Multivariable models will be constructed using variables measured prior to hospital discharge as independent variables and emergency hospital readmission within 90 days as the outcome variable. These variables will be derived from the linked databases. Multivariable regression approaches, including Cox regression or logistic regression (depending on dependent outcome variable), will be used to identify factors associated with increased readmission risk. The effect of death on the readmission outcome in models will be explicitly considered. We will identify patterns of healthcare contact after surviving critical illness by reporting emergency healthcare contact (emergency hospital readmission) and elective healthcare contact (outpatient attendance, elective/day case hospital admission). The number and type of variables of interest will be limited by the data available, which include patient demographics, characteristics of the ICU admission and data on some chronic pre-existing conditions that are available or can be derived from existing data sets. Although the variables associated with readmission, the strength of their association and their prevalence or incidence within the population will be derived using deductive analysis, the aim is to generate potential or proxy items for integration into an overall construct or taxonomy that describes reasons for readmission.

*RQ2: Can ICU survivors at highest risk of subsequent readmission be identified before hospital discharge?* Predictive models will be developed and validated in separate cohorts and model performance assessed. Derivation will be undertaken with a 70% sample using bootstrapping as an internal validation method which avoids model overfitting, and validation performance assessed in the remaining 30% of cases. If the model has sufficient predictive performance, it will be used to triage patients into risk groups at the time of hospital discharge, and the characteristics of patients in the highest risk groups described.

The anticipated outputs of the quantitative study will be: identification of key risk factors for unplanned early readmission; and a predictive model to identify and potentially triage patients prior to hospital discharge. We also plan to explore model performance when varying the timing of readmission cut-off over 1–12 months to identify the optimum evaluation period.

### Qualitative study

RQ3: What are the reasons early unplanned readmission occurs?

#### Participants

We will recruit up to 60 participants (30 patients and 30 family/carers) from three Scottish Health Board areas (NHS Lothian, NHS Tayside, NHS Fife). Patients will be identified from ICU databases and local hospital information systems by searching all hospital discharges following ICU admissions for patients who required unplanned readmission within 3 months of hospital discharge. We aim to interview participants once during the 3 months following their early unplanned readmission.

Inclusion criteria will comprise patients and their family members/carer who received invasive mechanical ventilation for ≥48 hours during their primary ICU admission; have been readmitted into hospital as an emergency within 90 days of subsequent hospital discharge; live within the nominated health boards (Fife, Lothian, Tayside); are aged ≥18 years; and have the capacity to give informed consent. Relatives/carer participants of patients fulfilling the first three inclusion criteria will be eligible. Exclusion criteria will be patients who underwent organ transplantation; a primary neurological admission diagnosis (brain trauma, intracerebral bleed, stroke, Guillain-Barré syndrome); patients receiving palliative care; unable to speak English; and patients considered too ill to participate by their general practitioner (GP).

#### Recruitment strategy

Two strategies will be employed. First, patients will be retrospectively identified from cohorts managed in the ICUs during the preceding 6 months; second, current ICU admissions will be prospectively tracked to identify patients who are subsequently readmitted within 90 days of their primary ICU admission. A purposive sampling strategy will be used with the aim of recruiting patients with a range of age, gender, social deprivation status, social situation (living alone vs partner), primary ICU admission diagnosis, illness severity, duration of ICU stay, mechanical ventilation during primary ICU admission and pre-existing comorbidity.

For retrospective recruitment, we will contact GPs to ascertain the status of the patient and send a letter of invitation from a locally appropriate ICU consultant, a participant information sheet and a consent form. Participants will be invited to contact the dedicated research associate (RA). One week after the letter of invitation has been sent out to the potential participant, a follow-up phone call will be made to ascertain whether the potential participant requires any further information and discuss possible participation. For prospective recruitment, potential participants will be identified at the time of discharge from intensive care and followed weekly for readmission to hospital within 90 days of discharge using hospital databases. Where possible, potential participants will be approached by research staff while in hospital; participants discharged home will be contacted by letter and phone call in the community.

#### Interviews

Interviews will take place in the patient or relative/carer's own home or in clinical research facilities, according to their preferences. Interviews will be with individual patients alone, with relative/carer alone or with a relative/patient together depending on participants' preference. Interviews will be semistructured using a preliminary taxonomy informed by the literature review and observations from the expert panel. Participants will be invited to discuss, in depth, those items or issues which they felt contributed to their (or their family member's) acute hospital readmission. They will subsequently be asked to group contributory items together and to rank them in order of importance. Participants will be invited to identify strategies or interventions that they feel may have prevented their readmission. The interview guide will be modified based on new themes identified during on-going analysis and/or triangulation with data from the quantitative study.

Information will be supplemented by several structured questionnaires that will provide data on perceived social support (Medical Outcomes Study Social Support (MOS-SS) Survey)^40^ and multimorbidity (Functional Comorbidity Index (FCI)).^41^ These will be completed with patients after the qualitative interviews have been conducted. In addition, we will ask relatives/carers to complete the Modified Caregiver Strain Index (MCSI),^42^ a widely used and validated structured questionnaire scale composed of 13 questions related to physical and emotional health, family finances, social interactions, time demands and employment. The MCSI tool may inform caregiver issues that may contribute to readmission risk. Finally, we will ask patients to numerically rate (0–10) the importance of factors identified in the literature review for their readmission (table 1).

**Table 1:** Items that patients and carers are asked to rank in importance to assist interpretation of their experience

From patient/carer perspective, how much did the following play in the acute readmission on a scale 0–10 (0=none, 10=very large part)?	Healthcare support from GP in community Healthcare support from nurses in community Psychological issues being addressed Support in community from social services Support from physiotherapy Social support (explain) from family/friends Communication between hospital and GP after discharge Communication between hospital and family Quality of information provided to myself and family on what to expect/do after discharge back home Any other factors
From patient/carer perspective, how would you grade the following in terms of supporting you at home after hospital discharge on a scale 0–10 (0=very weak, 10=very strong)?	Healthcare support from GP in community Healthcare support from nurses in community Support from physiotherapy Support in addressing psychological issues Support in community from social services Social support (explain) from family/friends Communication between hospital and GP after discharge Communication between hospital and family Quality of information provided to myself and family on what to expect/do after discharge back home

GP, general practitioner.

#### Data analysis

Interviews will be digitally recorded and transcribed verbatim. The transcribed data will be entered into a qualitative data analysis tool (NVIVO V.10). Thematic analysis will be used to code and organise data into common themes and explore individual, systemic, patient-centred and clinical factors and also cumulative factors which participants identified as contributing to acute hospital readmission, including their relative importance. The initial interview transcripts will be read independently and coded independently by a subgroup of two PROFILE team members before meeting together to discuss their interpretation and produce reports of their own determination of themes, subthemes and issues within and across the data for discussion. Analysis will occur throughout the sequential interviews, iteratively exploring and refining items until data saturation is achieved (the point at which no new themes emerge). We will explore these factors between participant and family member/carers dyads and across all accounts (within and cross-case analysis). We will adopt an inductive approach without an a priori analytical framework, which will also be informed by emerging themes from the quantitative data.

### Data integration strategy

#### Quantitative data

We anticipate that modelling will provide associations between the available exposure variables and the probability of an unplanned readmission occurring. The prevalence of these factors among patients with/without unplanned readmission and the strength of association will be summarised. We expect these factors will be limited to patient demographic (eg, age, gender, Scottish Index of Multiple Deprivation, rurality), chronic illness (Charlson comorbidities) and acute illness characteristics (ICU admission diagnosis, illness severity at ICU admission, duration of index ICU and/or hospital stay) due to the nature of the data sets. Quantitative models will lack information on potentially important drivers such as social situation, health literacy, discharge planning and follow-up, high-risk treatments and caregiver support issues.

#### Qualitative data

We anticipate qualitative data will be summarised in major themes or categories that will emerge from the analysis. We anticipate a more detailed understanding of psychosocial, behavioural and sociodemographic factors and other issues will emerge. The patient questionnaires and self-ranking of readmission drivers may be informative in helping to quantify patient observations of what influenced their readmission.

#### Triangulation and integration of data

The multidisciplinary researcher group will have a series of meetings to integrate the two data sources into a preliminary construct and taxonomy for readmission drivers. Our approach will be to check and compare each theme/item/predictor that emerged from the qualitative data with the quantitative data and explore concordance and discordance systematically. This process will be repeated using each predictor in the quantitative analysis compared with the qualitative data. Both data forms will be considered of equal importance in the triangulation. The purpose of triangulation will be to enrich understanding of data derived from both sources, refute or confirm findings when comparing the two sources, and to explain unexpected or discordant findings.^43^ The group will use a collective brainstorming approach to explore the following questions about each potential readmission driver: Is this theme captured/represented in the quantitative (qualitative) data? If yes, is there concordance? If yes, is there discordance and what is the reason for this? What conclusions can be drawn about prevalence as a readmission driver? What conclusions can be drawn about importance as a readmission driver?

Consensus will be sought from the group, and clearly documented for each theme within the qualitative data using this structure. The evidence supporting the group conclusions, drawn from both data sources, will be summarised in the form of a matrix or diagram.

#### Preliminary construct

Through group consensus, we will generate the initial construct in the form of tables, diagrams or checklists according to the findings. The construct will aim to describe the following:

1. A taxonomy/checklist: this will capture factors/drivers/processes that appear to explain readmission, the possible mechanism, prevalence and strength/importance.
2. Potential mechanisms, pathways and interactions: this will capture and describe how individual risk factors or processes operate to result in readmission, and how different risk factors may interact to mediate risk. We will aim to provide a theoretical construct that could underpin and/or justify the development of a complex healthcare intervention designed to alter risk. The aim will be to provide the basis of a toolkit or intervention package.

We will use case histories from the qualitative interviews to support the conclusions of the integration and preliminary construct.

### Validation

We will undertake two forms of validation (and refinement) to the preliminary construct.

1. Focus groups across six Scottish health boards with patients (and their carers), who required unplanned acute hospital readmission: We will screen patients from six ICUs and approach them using the same strategy defined for the interview-based component of the study. A semistructured topic guide will be developed based on the preliminary construct to facilitate discussion of the factors, themes and drivers identified. Concordant and discordant views will be sought, and any additional missing factors explored. Separate focus groups will be undertaken in each health board region comprising 6–10 participants, and will be recorded and transcribed for analysis. Data from focus groups will be thematically analysed and systematically evaluated against the preliminary construct, and a summary report written highlighting concordance, discordance and additional insights. These will be reviewed by the multidisciplinary researcher group, and revisions made, with documentation of justifying data.
2. Review with external expert panel: The revised construct and report will be presented to the external panel, together with relevant evidence. The final construct will be agreed on the basis of this discussion and expert review.

## Ethics and dissemination

The quantitative analyses have received waiver from the ethics committee, but were approved by the Privacy Advisory Committee. The qualitative studies received ethical approval from the regional ethics committee (South East Scotland Research Ethics Committee 02 (reference number 14/SS/1032)). Our dissemination strategy includes a formal knowledge exchange meeting with multiple stakeholders at the end of the project, as well as presentations at meetings and publications.

## Current status

The project started on 1 June 2014. Recruitment to the individual interview study was completed in September 2015, and analysis completed in January 2016. The analysis of the quantitative data was completed in January 2016. Data triangulation occurred during January–March 2016. The validation focus groups are currently ongoing (April–June 2016). The project will finish in October 2016.

## Comment

PROFILE will be the first study to explore in detail the reasons that so many patients who survive critical illness require emergency hospital readmission within a few weeks of hospital discharge. The value of readmission as a quality indicator in other healthcare populations is controversial, particularly as it has been used as a quality indicator and linked to financial penalisation in some healthcare systems.^44^ Proponents argue that system failure is a major cause of unplanned readmission, either because patients were discharged from hospital before adequate recovery, they suffered late complications or their preparation for community living was inadequate, for example, in terms of ongoing support from community services. Opponents argue that non-modifiable factors may dominate readmission events, such as physical frailty or impairments, which may vary between geographical regions based on factors such as socioeconomic demographics. Some evidence also suggests that populations that are prevalent among critical care survivors, especially those admitted for sepsis, are more likely to require readmission with related complications such as new infections.^45^

PROFILE will provide detailed information about the many possible factors that could plausibly contribute to readmission. Strengths are the use of mixed methods that include population-level data and a large number of interviews with patients and carers who have experienced the event of interest. This mixture of deductive and inductive approaches will increase the chance of capturing all important issues, and understanding how they may interact. By generating qualitative and quantitative data concurrently and regularly sharing findings we will maximise the value of each data source. The inclusion of focus groups with stakeholders, patient/public and experts, will help validate the findings and ensure that major issues have not been missed. Limitations include the restriction to Scotland, so external generalisability to other geographical regions and healthcare systems may be uncertain. It may also be difficult to fully explore how the patient surviving critical illness differs from other patient groups because we are not including patient populations who did not require intensive care. Finally, although our study will inform the development of possible healthcare or social interventions that might prevent readmission, future studies will be needed to test their effectiveness.
